# Epilepsy and migraine: a diagnostic and therapeutic challenge

**DOI:** 10.3389/fphar.2025.1649543

**Published:** 2025-08-29

**Authors:** Angelo Pascarella, Oreste Marsico, Domenico Abelardo, Roberta Cutellè, Alessandro Bulgari, Cataldo Mummolo, Anna Mammì, Vittoria Cianci, Umberto Aguglia, Edoardo Ferlazzo, Sara Gasparini

**Affiliations:** ^1^ Department of Medical and Surgical Sciences, Magna Græcia University of Catanzaro, Catanzaro, Italy; ^2^ Regional Epilepsy Centre, Great Metropolitan “Bianchi-Melacrino-Morelli Hospital”, Reggio Calabria, Italy; ^3^ Department of Neuroscience, “Giovanni Paolo II” Hospital, Lamezia Terme, Italy; ^4^ Neurology Unit, Great Metropolitan “Bianchi-Melacrino-Morelli” Hospital, Reggio Calabria, Italy

**Keywords:** antiseizure medications, seizure, aura, headache, hemiplegic migraine, migralepsy

## Abstract

Migraine and epilepsy are two common, chronic, disabling, paroxysmal neurological disorders. A growing body of evidence from epidemiological, genetic, neurophysiological, and clinical research suggests a complex, bidirectional association between them. Migraine prevalence in epilepsy patients ranges from 8% to 23%, while the reverse is noted at 1%–17%. Both disorders are underpinned by cortical hyperexcitability, dysfunctional neurotransmission, and impaired ion homeostasis. Shared genetic mutations, particularly in genes encoding ion channel subunits such as CACNA1A, SCN1A, and ATP1A2, further support a common channelopathy model. Cortical spreading depression, the electrophysiological substrate of migraine aura, and paroxysmal depolarization shift, a hallmark of epileptic activity, share converging features, including neuronal depolarization, potassium accumulation, glutamate release, and eventual firing suppression. Glial dysfunction, glutamatergic excitotoxicity, and mitochondrial deficits are additional unifying elements. Clinically, the differential diagnosis between migraine with aura and focal seizures remains challenging due to overlapping sensory, visual, and autonomic symptoms. Rare phenomena including ictal epileptic headache, postictal headache, and migraine-triggered seizures further complicate the clinical spectrum. Additionally, certain epilepsy syndromes, such as childhood epilepsy, are strongly associated with migraine. Early recognition of comorbidity is crucial for appropriate management, as tailored treatment strategies may improve outcome. Several antiseizure medications, including topiramate, valproate, lamotrigine, and perampanel, also demonstrate efficacy in migraine prophylaxis. Moreover, non-pharmacological approaches such as ketogenic diet, vagus nerve stimulation, and transcranial magnetic stimulation provide further evidence of a shared neurobiological substrate. This review explores the epidemiological, pathophysiological, and clinical intersections between migraine and epilepsy, a frequent and clinically relevant dilemma. Accurate differentiation is urgently needed to avoid therapeutic delays or inappropriate interventions, given their phenotypic mimicry. In addition, it highlights therapeutic implications driven by overlapping molecular mechanisms. Ongoing research is needed to further elucidate this relationship.

## 1 Introduction

Migraine and epilepsy represent two of the most prevalent and disabling chronic neurological disorders, both characterized by recurrent, transient, and paroxysmal disturbances of cerebral function (Haut et al., 2006). Although traditionally regarded as distinct conditions with different clinical profiles and diagnostic criteria, growing evidence from epidemiological, genetic, and neurophysiological studies now indicate a complex and bidirectional relationship (Andermann, 1987; Cianchetti et al., 2017a; Bauer et al., 2021; Paungarttner et al., 2024). Epidemiological data consistently demonstrate a bidirectional association, whereby individuals affected by one condition exhibit an increased risk of developing the other. This pattern points to a potential shared etiological substrate, suggesting the existence of a link that goes beyond mere coincidence (Nye and Thadani, 2015; Keezer et al., 2015; Bauer et al., 2021).

At the molecular level, converging findings implicate common genetic mutations, particularly in ion channel-related genes such as CACNA1A, SCN1A, and ATP1A2, in the pathogenesis of both disorders. These mutations provide a biological basis for this association, supporting the hypothesis of a shared channelopathy framework (Gotra et al., 2021; Bauer et al., 2021; Paungarttner et al., 2024). Furthermore, both conditions are marked by cortical hyperexcitability, dysfunctional neurotransmitter systems, and abnormal neuronal network synchronization (Papetti et al., 2013, Mantegazza et al., 2021; Bauer et al., 2021; Demarquay and Rheims, 2021). Clinically, migraine and epilepsy may present with overlapping ictal symptoms, including visual auras, sensory disturbances, and autonomic manifestations, which often pose diagnostic challenges, principally in childhood and adolescence (Nye and Thadani, 2015; Garg and Tripathi, 2021). In addition, several antiseizure medications (ASMs) have demonstrated efficacy in migraine prophylaxis, highlighting potential commonalities in treatment response and neurochemical targets (Pelzer et al., 2023; Demarquay and Rheims, 2021). Despite these insights, the precise nature of the relationship between migraine and epilepsy (whether comorbid, causally linked, or reflecting distinct but overlapping pathologies) remains incompletely understood. Recognizing this overlap is essential to avoid misdiagnosis and to ensure an appropriate management and treatment approach.

**FIGURE 1:**
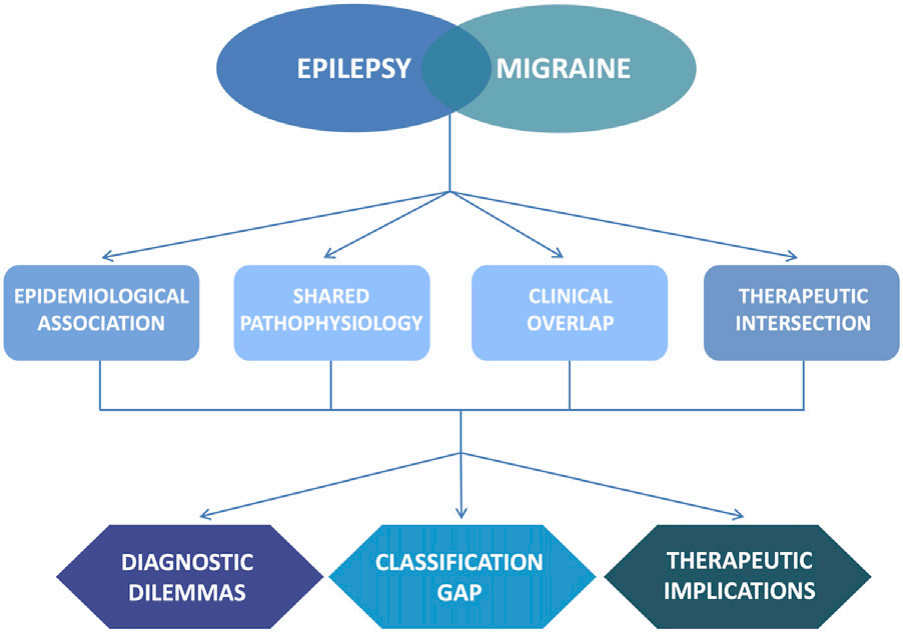
Overview of the main purpose and key concepts of the review. The figure summarizes the complex relationship between epilepsy and migraine. It illustrates the key topics discussed, including epidemiological, pathophysiological, clinical, and therapeutic aspects. It also highlights the clinical impact and the remaining knowledge gaps that require further research.

This review provides a comprehensive synthesis of current literature on the migraine-epilepsy association, focusing on shared pathophysiology, clinical overlaps, diagnostic dilemmas, classification gap and therapeutic implications (Figure 1). We also highlight persistent gaps in understanding causal links and optimizing cross-disciplinary treatment strategies.

## 2 Methods

For this narrative review, source references were identified using PubMed and Google Scholar until April 2025, by means of the terms “epilepsy,” “seizure,” “migralepsy” in various combinations with “migraine,” “headache.” No date limits were applied; the search included studies from database inception. Only articles in English language were selected, which may introduce language bias. As this is a narrative review, no formal quality assessment tool was applied. Search results were reviewed manually, and original studies, systematic reviews and meta-analyses were selected based on importance, originality, quality, and relevance to the purpose of this review.

## 3 Results

### 3.1 Epidemiological data

Numerous studies have reported a bidirectional association between epilepsy and migraine. The prevalence of migraine among individuals with epilepsy ranges from 8.4% to 23%, while the prevalence of epilepsy among migraineurs is estimated between 1% and 17% (Matias-Guiu et al., 1992; Brodtkorb et al., 2008; Toldo et al., 2010; Keezer et al., 2015; Duko et al., 2020; Atalar et al., 2022). The wide variability in reported prevalence likely reflects differences in study design, diagnostic criteria, and population characteristics. However, these figures may be higher than those observed in the general population, where migraine affects approximately 14%–15% of subjects and epilepsy 0.5%–1% (Hauser et al., 1991; Lipton and Bigal, 2005; Fiest et al., 2017; Steiner and Stovner, 2023). A 2014 meta-analysis of population-based studies found a 52% increased prevalence of migraine in individuals with epilepsy compared to controls (pooled risk [PR]: 1.52; 95% CI: 1.29–1.79), and a 79% increased prevalence of epilepsy among individuals with migraine (PR: 1.79; 95% CI: 1.43–2.25) (Keezer et al., 2015). A more recent study using random-effects models reported an 80% increase in the lifetime prevalence of each condition when the other is already present (OR/RR: 1.80; migraine in epilepsy: 95% CI: 1.35–2.40; epilepsy in migraine: 95% CI: 1.43–2.25) (Wu and Zhuang, 2024). Additional studies report that up to 79% of individuals with epilepsy experience headaches, with migraine occurring in up to 25% and tension-type headache in up to 40% of cases. Notably, women are more likely than men to report migraine (Begasse de Dhaem et al., 2019; Whealy et al., 2019; Bauer et al., 2021).

However, despite accumulating evidence supporting a relationship between epilepsy and migraine, not all data are consistent. A large population-based cohort study (n = 65,407) found no significant difference in the prevalence of migraine (OR: 0.95; 95% CI: 0.68–1.33) or non-migraine headache (OR: 1.18; 95% CI: 0.93–1.50) between individuals with epilepsy and controls (Engstrand et al., 2024). Current evidence remains insufficient to confirm a definitive correlation between migraine and epilepsy (Hesdorffer et al., 2007; Brodtkorb et al., 2008). Moreover, the up-to-date epidemiological data remain limited by several methodological concerns including recall bias, reliance on self-reported data, use of unvalidated diagnostic tools (studies that used self-report questionnaires tended to show a stronger association), and potential diagnostic overshadowing. Furthermore, most available studies are cross-sectional and do not allow inference of causality (Keezer et al., 2015; Bauer et al., 2021; Engstrand et al., 2024).

Future prospective, longitudinal, and multicenter studies with standardized diagnostic criteria are essential to better define the nature and directionality of the epilepsy–migraine comorbidity. A clearer understanding of this association will enhance clinicians’ awareness of possible coexisting migraine and epilepsy, promote timely and appropriate screening and diagnosis, and support integrated, tailored treatment strategies, ultimately improving patient outcomes.

### 3.2 Shared pathophysiological mechanisms underlying epilepsy and migraine

Epilepsy and migraine, particularly migraine with aura, share striking similarities in their underlying pathophysiology. Both are characterized by episodic dysfunctions of neural excitability, and growing evidence from clinical observations, electrophysiological studies, genetic analyses, and molecular investigations supports their interconnectedness (Rogawski, 2008). Despite their distinct clinical expressions and temporal dynamics, a common foundation of cortical hyperexcitability and altered ion homeostasis suggests these two disorders exist on a pathophysiological continuum (Papetti et al., 2013; Mantegazza, 2018; Bauer et al., 2021, Paungarttner et al., 2024).

**FIGURE 2:**
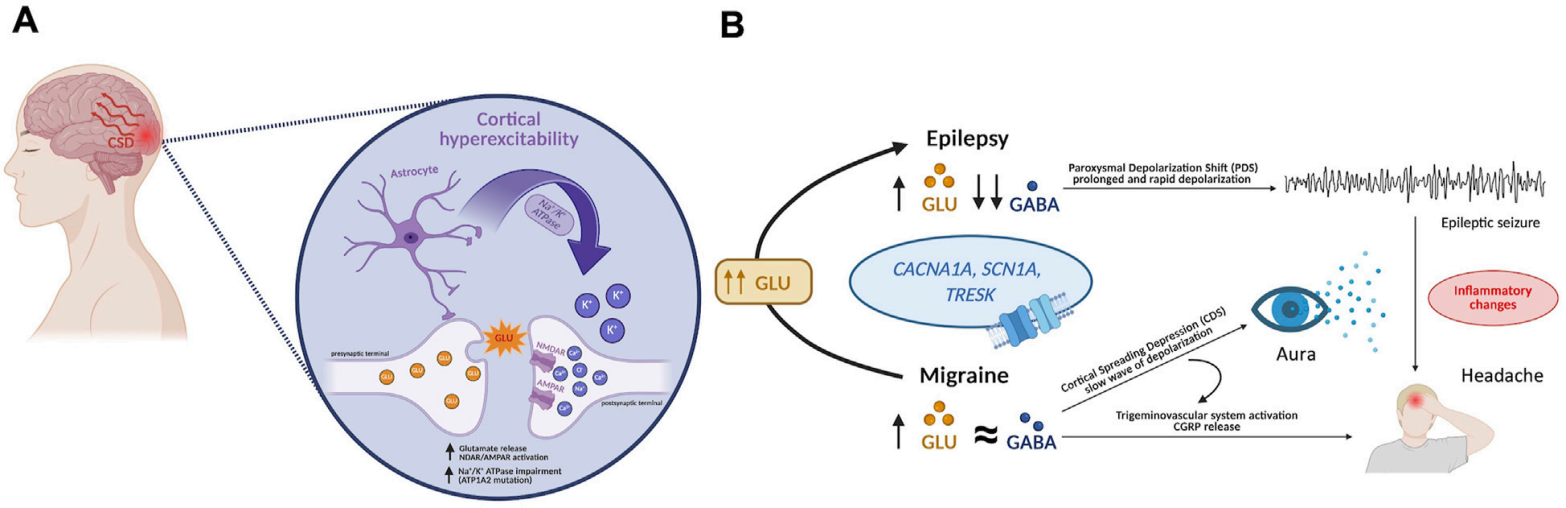
Insight into the shared pathophysiological mechanism of epilepsy and migraine. **(A)** Cortical spreading depolarization (CSD) is the main neurophysiological mechanism underlying migraine. It consists of a slowly propagating wave across the cortical network, likely triggered by cortical hyperexcitability. CSD is associated with elevated concentrations of potassium ([K^+^]. Astrocytes play a key role in maintaining extracellular K^+^ and glutamate (GLU) homeostasis via Na^+^/K^+^-ATPase activity. Mutations impairing astrocytic K^+^ uptake, disrupting ionic buffering, and leading to defective K+ clearance, enhances excitatory neurotransmission. This results in excessive glutamate release from presynaptic terminals and overactivation of postsynaptic NMDA and AMPA receptors. **(B)** In epilepsy, increased GLU levels and reduced GABAergic inhibition led to neuronal hyperexcitability, sustained by paroxysmal depolarization shifts (PDS), which culminate in epileptic seizures. In migraine, elevated GLU relative to GABA promotes CSD, which activates the trigeminovascular system and triggers the release of calcitonin gene-related peptide (CGRP), resulting in headache. CDS underlies the clinical manifestation of migraine aura (most commonly visual). Genetic mutations affecting ion channels (i.e., CACNA1A, SCN1A, TRESK) contribute to excitatory/inhibitory imbalance. Postictal headache may occur after seizures, likely due to seizure-induced vascular or inflammatory changes. Excessive glutamatergic transmission represents a pathophysiological mechanism linking these two neurological disorders (Created with BioRender.com).

At the heart of both epilepsy and migraine lies a disturbance in cortical excitability, stemming from an imbalance between excitatory and inhibitory neurotransmission. Although this imbalance manifests differently, it is a core feature of both conditions (Figure 2) (Laplante et al., 1983; Papetti et al., 2013). In epilepsy, seizures result from pathological, hypersynchronous neuronal discharges originating in hyperexcitable regions. When neuronal depolarization reaches a critical threshold, it triggers repetitive action potentials, leading to seizures. These can remain localized (focal seizures) or propagate through broader brain networks (generalized seizures) (Fisher et al., 2017). The key cellular electrophysiological hallmark of neuronal excitability of an epileptic focus is the paroxysmal depolarization shift (PDS), characterized by a prolonged membrane depolarization (up to 30 mV) lasting tens to hundreds of milliseconds, initially associated with high-frequency spiking, followed by progressive attenuation and suppression of neuronal firing, known as depolarization block (Matsumoto and Marsan, 1964; Mantegazza, 2018).

The precise pathophysiology of migraine is still an active area of research and is not yet fully elucidated. Historically, migraine was predominantly conceptualized as a primary vascular disorder. However, current research increasingly points towards neurogenic or neurovascular mechanisms. A compelling hypothesis suggests that neuronal network dysfunctions, leading to cortical hyperexcitability, may serve as the precipitating factor for migraine attacks (Goadsby et al., 2017; Charles, 2018). Migraine, especially migraine with aura, is associated with cortical spreading depression (CSD). CSD is a slowly propagating wave of neuronal and glial depolarization that moves across the cortex at a rate significantly slower than epileptic discharges (2–3 mm/min). This wave is preceded by a brief period of hyperactivity and followed by a prolonged suppression of synaptic activity and neuronal firing (Lauritzen, 1994). Experimental studies, specifically those involving familial hemiplegic migraine (FHM) mutations, have shown a lowered threshold for CSD induction, supporting a genetically determined increase in excitability in migraine (Nye and Thadani, 2015). The migraine aura, most commonly visual but also presenting as sensory, motor, or language disturbances, is the clinical correlate of CSD. CSD has also been consistently shown to activate the trigeminovascular system in migraine. This activation triggers a cascade of events, notably the release of calcitonin gene-related peptide (CGRP), which then plays a central role in initiating the headache phase through vasodilation and neurogenic sterile meningeal inflammation (Hadjikhani et al., 2001; Bolay et al., 2002; Zhang et al., 2011; Romero-Reyes and Akerman, 2014; Goadsby et al., 2017). While epilepsy lacks a direct equivalent to this CSD-driven headache mechanism, postictal headache may occur after seizures, potentially due to seizure-induced vascular or inflammatory changes, or possibly even CSD-like events triggered by the seizure activity itself (Parisi et al., 2008; Bastany et al., 2020).

A critical point of convergence between the two conditions is ionic dysregulation, specifically the accumulation of extracellular potassium ([K^+^]_e_). During both seizures and CSD, [K^+^]_e_ increases due to enhanced neuronal activity and impaired clearance. However, the dynamics differ: [K^+^]_e_ typically rises to 8–10 mM in seizures, while it often exceeds 20 mM during CSD (Chang et al., 2018; Menyhárt et al., 2018). This more abrupt and sustained elevation in CSD contributes significantly to neuronal depolarization and subsequent activity suppression (Basarsky et al., 1998; Larrosa et al., 2006). Similarly, glutamatergic excitotoxicity links the two conditions at the synaptic level. Glutamate is released into the synaptic space in both conditions, activating NMDA and AMPA receptors and further enhancing depolarization. These shared ionic disturbances promote pathological excitation and predispose to network instability. Excessive glutamate release activates ionotropic receptors, leading to intracellular Ca^2+^ overload, activation of degradative enzymes, oxidative stress, and, in severe cases, neuronal death. In epilepsy, this mechanism sustains ictal propagation and contributes to network reorganization. In migraine, glutamate-induced depolarization and excitotoxic signaling may trigger CSD and sensitize nociceptive pathways, particularly in the trigeminovascular system (Van Harreveld and Fifková, 1973; Nellgård and Wieloch, 1992; Mei et al., 2020). Astrocytes are crucial for regulating [K^+^]_e_ and glutamate through Na^+^/K^+^-ATPase and excitatory amino acid transporters (EAATs). Loss-of-function mutations in ATP1A2, for example, reduce astrocytic K^+^ uptake, impairing ionic buffering and promoting a hyperexcitable extracellular environment. In epilepsy, downregulation of EAATs and altered astrocyte gap junction coupling hinder glutamate clearance and K^+^ diffusion, exacerbating seizure propensity. Astrocytic calcium signaling and dysfunction in glial-neuronal communication are increasingly recognized as active contributors to cortical excitability in both migraine and epilepsy (Leo et al., 2011; Ferrari et al., 2015; Bøttger et al., 2016; Mantegazza, 2018).

Mitochondrial dysfunction exacerbates energy failure in both disorders (Goadsby et al., 2017). ATP depletion impairs ion pumping and synaptic cycling, facilitating membrane depolarization and lowering thresholds for CSD and seizures. Clinical syndromes like Mitochondrial Encephalopathy with Lactic Acidosis and Stroke-like episodes (MELAS) strikingly illustrate the convergence of migraine and epilepsy due to impaired oxidative phosphorylation. Patients with MELAS are susceptible to both migraine with aura and epileptic seizures, with pathological studies revealing cytochrome oxidase deficiency, particularly within the smooth muscle and endothelial cells of meningeal blood vessels. While cerebral ischemic lesions are prominent in posterior regions (consistent with visual aura) it remains unclear if these patients experience altered CSD or the precise mechanism of their seizures (Nye and Thadani, 2015; Liao et al., 2018; Kuwajima et al., 2019).

Stroke-like Migraine Attacks after Radiation Therapy (SMART syndrome) is a rare condition occurring several years after radiotherapy; patients may experience headache with migraine features, but a high proportion also have epileptic seizures (Ferlazzo et al., 2018). The physiopathology is not entirely known, but radiation-induced microvascular damage may play a role. An alternative hypothesis involves post-radiation neuronal dysfunction with impairment of the trigeminovascular system or a lowered threshold for cortical spreading depression (Patel et al., 2020).

Genetic channelopathies offer additional evidence of shared pathophysiology. Genetic studies have identified mutations in voltage-gated ion channels (such as CACNA1A, SCN1A, and ATP1A2) which regulate neuronal excitability (Rogawski, 2008; Nye and Thadani, 2015; Bauer et al., 2021; Paungarttner et al., 2024). Mutations in CACNA1A (encoding the α1A subunit of the P/Q-type calcium channel) are implicated in familial hemiplegic migraine type 1 (FHM1) and also confer susceptibility to epilepsy. These mutations enhance calcium influx and glutamate release (Ophoff et al., 1996; Tottene A et al., 2002). Similarly, mutations in ATP1A2 (responsible for FHM2) and SCN1A (associated with Dravet syndrome and other epileptic syndromes) disrupt potassium and sodium channel function, respectively, lowering thresholds for both CSD and epileptiform activity (Catterall, 1998; Dichgans et al., 2005; Kasperaviciute et al., 2013; Friedrich et al., 2016). These mutations are found in both familial hemiplegic migraine (FHM) and epilepsy syndromes, reinforcing the concept that migraine and epilepsy exist on a continuum of neuronal instability rather than as entirely separate entities (Hasırcı Bayır et al., 2021). Beyond genetic mutations, post-translational modifications and altered expression of ion channels also contribute to acquired hyperexcitability. Additionally, genes like KCNQ2 (involved in potassium channel function), TRESK (coding for another potassium channel), PRRT2 (implicated in Regulation of Ca^2+^ - mediated neurotransmitter release and voltage-gated ion channels) have been linked to both benign familial neonatal seizures and some forms of migraine (Ebrahimi-Fakhari et al., 2015; Royal et al., 2019; Aiba and Noebels, 2021; Schreiber et al., 2022). Notably, loss-of-function mutations in TRESK channels increase trigeminal sensory neuron excitability, contributing to migraine susceptibility (Royal et al., 2019), and experimental evidence suggests that TRESK dysfunction may also enhance hippocampal neuronal excitability and seizure severity, further supporting its dual role in migraine and epilepsy pathophysiology (Huang et al., 2021).

### 3.3 Clinical overlap and classification

From a clinical perspective, differentiating between migraine aura and focal seizures can sometimes be a significant challenge for neurologists. Both conditions manifest as paroxysmal episodes, often presenting with overlapping neurological disturbances that may precede or accompany the main clinical event (headache in migraine, ictal symptoms in epilepsy). These shared features encompass visual phenomena, sensory disturbances, motor symptoms, language impairments, and alterations in awareness (Flanagan and Ghose, 2000). The key to accurate diagnosis lies primarily in a detailed patient history (Nye and Thadani, 2015).

Visual symptoms are the most frequently overlapping features. In migraine with aura, patients typically describe negative visual symptoms, such as scintillating scotomas or zig-zag figures (fortification spectra), with gradual onset and a duration of 5–60 min, followed by headache (Schott, 2007). In contrast, visual auras in occipital lobe seizures are usually shorter (a few seconds to 2–3 min), have a sudden onset, are more stereotyped, and are rarely followed by headache (Panayiotopoulos, 1999a). Epileptic visual auras often affect a single hemifield and consist of bright, flashing lights, colorful circular patterns, and rapid dynamics (Panayiotopoulos, 1999b). Thus, the onset pattern and temporal evolution are key elements in differential diagnosis. A useful tool in this setting is the Visual Aura Rating Scale (VARS), which has been validated to distinguish visual migraine aura from other transient visual disturbances, with a sensitivity of 91% and specificity of 96% (Eriksen et al., 2005). Sensory symptoms like paresthesia, numbness, or tingling can be reported in both conditions. In migraine aura, these symptoms often begin in one hand and spread slowly to the arm and face over several minutes, sometimes even crossing to the other side (Schachter et al., 1995). Conversely, focal sensory seizures tend to involve more abrupt, focal, and stereotyped paresthesia, often restricted to a specific body part with rapid or no progression.

Similarly, olfactory, gustatory, or gastric sensations associated with temporal lobe epilepsy auras last only for seconds, whereas nausea and other sensory disturbances linked to a migraine attack can persist for hours or days (Klass and Westmoreland, 1985). Hemiplegic migraine may clinically mimic Todd’s paresis, as both conditions present with prolonged unilateral weakness that can last hours to days. Both may show EEG slowing contralateral to the weakness. Clinical context, associated features, and family history often aid in differential diagnosis (Russell and Ducros, 2011; Di Stefano et al., 2020). Language disturbances (dysphasia or aphasia) may also occur in both conditions. In migraine, aphasia typically appears during the aura and resolves within an hour. In focal temporal lobe seizures, speech may be impaired during ictal and postictal phases, often with accompanying automatisms or confusion (Nye and Thadani, 2015).

A confusional state, marked by disorientation, psychomotor agitation, and occasionally aggressive behavior lasting several hours, along with transient EEG slowing, can sometimes be an unusual picture of juvenile migraine (Emergy, 1977). However, it is essential to distinguish this presentation from psychiatric disorders, post-ictal state and non-convulsive status epilepticus (Flanagan and Ghose, 2000).

Headache itself may also occur as a symptom of epilepsy, manifesting in various temporal relationships with a seizure. The features may mimic primary headache disorders, particularly migraine, posing significant diagnostic challenges even for experienced clinicians. Headaches associated with seizures are classified as: a) pre-ictal, when headaches manifest <24 h before a seizure and persist until its onset; b) ictal, when pain is the sole or predominant seizure manifestation and emerge concurrently with the seizure itself; c) post-ictal, if headache begin <3 h of seizure termination and typically resolve spontaneously within 72 h (Bauer et al., 2021; Paungarttner et al., 2024).

While the International League Against Epilepsy (ILAE) does not explicitly classify seizures by their headache overlap, the International Classification of Headache Disorders, 3rd edition (ICHD-3) acknowledges the clinical overlap between the two conditions and directly addresses this significant clinical intersection by providing a specific section on headache disorders attributed to epileptic seizures (Headache Classification Committee of the International Headache Society, 2018; Bauer et al., 2021). The ICHD-3 includes distinct diagnostic categories for seizure-related headaches: migraine aura-triggered seizure (Section 1.4.4), ictal epileptic headache (Section 7.6.1) and post-ictal headache (Section 7.6.2) (Headache Classification Committee of the International Headache Society, 2018). A classification of pre-ictal headache remains absent from the ICHD-3, even if comments section explicitly advocates for further research to establish the existence, prevalence, and precise features of these headaches.

While pre-ictal headache is a recognized phenomenon in epilepsy (Leniger et al., 2001; Förderreuther et al., 2002; Karaali- Savrun et al., 2002; Yankovsky et al., 2005), it currently lacks formal classification within the ICHD-3. Despite this, the ICHD-3 in its comments section explicitly advocates for further research to clarify the existence, prevalence, and precise features of these headaches. Studies report pre-ictal headaches in 1%–10% of epilepsy patients, often presenting as migraine-like (30%–60%) or tension-type (around 20%) (Leniger et al., 2001; Förderreuther et al., 2002; Karaali- Savrun et al., 2002; Yankovsky et al., 2005; Wang et al., 2014b; Hofstra et al., 2015; Mainieri et al., 2015; Mameniškienė et al., 2016; Seo et al., 2016; Çilliler et al., 2017; Mutlu, 2018; Salma et al., 2019). However, many reports lack simultaneous EEG documentation demonstrating the absence of ictal discharges during the headache (which is a key diagnostic hurdle and mandatory requirement for definitively classifying a headache as pre-ictal) leaving a gap in the unequivocal demonstration of purely pre-ictal phenomena. A specific video-EEG study identified pre-ictal headache in 3% (25 of 831) of individuals with epilepsy without concurrent epileptic discharges on EEG during the headache, ultimately reclassifying five of these cases as “headache as a seizure aura,” which, by definition, falls under ictal epileptic headache (Kim et al., 2016). This highlights the subtle yet crucial distinctions that demand robust neurophysiological correlation for accurate classification.

Migraine-triggered seizure (previously known as “migralepsy”) is defined in ICHD-3 as a seizure that occurs during or within one hour after a migraine aura (Lennox and Lennox-Buchthal, 1960; Headache Classification Committee of the International Headache Society, 2018). This rare diagnosis requires the migraine aura to fulfil criteria for migraine with aura and the seizure to follow closely (Sforza et al., 2021). The clinical validity of migralepsy remains a subject of considerable debate (Maggioni et al., 2008; Verrotti et al., 2011; Belcastro et al., 2011; Hartl et al., 2015). Many reported cases likely represent misdiagnoses, with either seizures being mistaken for migraine or migraine misinterpreted as epileptic events (Panayiotopoulos, 1999a; Sances et al., 2009). A review by Sances et al. examining 50 previously reported cases of migralepsy found that only two actually satisfied the stringent definition set forth by the ICHD-II. This low concordance highlights a critical issue: many cases historically labeled as migralepsy likely represent either epileptic seizures misdiagnosed as migraine or, conversely, migraine aura mistaken for epileptic events (Sances et al., 2009). This diagnostic ambiguity is particularly pronounced in pediatric epilepsy syndromes such as Gastaut and Panayiotopoulos syndromes, both of which share clinical features with migraine, significantly complicating accurate differentiation (Kasteleijn-Nolst Trenité and Parisi, 2012).

Ictal epileptic headache (IEH) is defined as a headache that constitutes the sole or predominant manifestation of a focal epileptic seizure, accompanied by simultaneous epileptiform EEG activity (Parisi et al., 2012b; Headache Classification Committee of the International Headache Society, 2018). According to the ICHD-3 criteria, the headache must begin concurrently with the seizure and resolve immediately after its termination (Headache Classification Committee of the International Headache Society, 2018). A lateralized headache ipsilateral to the ictal discharge reinforces diagnostic suspicion. Subtle motor, sensory, or autonomic features may sometimes accompany the headache. When headache is the only symptom, distinguishing IEH from primary headache disorders is particularly challenging (Parisi et al., 2015; Headache Classification Committee of the International Headache Society, 2018; Parisi et al., 2019). IEH is considered rare but likely underrecognized. It occurs across all age groups and shows no sex predilection (Cianchetti et al., 2017b; Parisi et al., 2019). Clinical presentation is heterogeneous and frequently mimics primary headache disorders such as migraine or tension-type headache. Pain may be localized or diffuse and often lacks a consistent correlation with structural abnormalities or specific epileptogenic foci. This variability, together with often inconclusive scalp EEG results, significantly complicates diagnosis (Parisi et al., 2012a; Cianchetti et al., 2017a; Salma et al., 2019; Bauer et al., 2021). In a cohort of 831 individuals with epilepsy undergoing video-EEG monitoring for peri-ictal headaches, six patients experienced headache as the sole seizure manifestation, with concurrent epileptiform discharges. These brief episodes (<35 s) were classified as auras and fulfilled criteria for IEH (Kim et al., 2016). In some cases, intracranial electrodes are necessary to detect deep or insular epileptogenic zones (Siegel et al., 1999; Fanella et al., 2015). While episodes typically last seconds to minutes, prolonged headaches may occur, particularly in nonconvulsive status epilepticus, resolving only after intravenous ASM administration (Belcastro et al., 2011). Some authors have proposed including response to ASMs as a diagnostic criterion, but interindividual pharmacodynamic variability limits its utility (Parisi et al., 2019; Bauer et al., 2021). Given its potential to mimic primary headache disorders and the need for prompt management to prevent status epilepticus, a timely and accurate identification of IEH is crucial to avoid unnecessary investigations and treatments often initiated in acute settings (Parisi et al., 2012a). Pathophysiologically, IEH likely results from epileptic activation of pain-sensitive cortical and subcortical regions—especially the insula, anterior cingulate cortex, and operculo-insular areas—leading to stimulation of the trigeminovascular system (Afif et al., 2008; Parisi et al., 2012a; Cianchetti et al., 2017b). Some episodes may represent autonomic seizures involving deeper networks such as the hypothalamus or medial temporal lobe (Belcastro et al., 2011; Parisi et al., 2012a; Parisi, 2015). Cortical spreading depression triggered by subclinical epileptic activity has also been proposed as a mechanistic link between IEH and migraine-like features (Parisi et al., 2008; Parisi, 2009).

A rare subtype, hemicrania epileptica, is characterized by strictly unilateral headache on the same side as the ictal EEG discharge (Headache Classification Committee of the International Headache Society, 2018). Its existence remains debated, as ictal EEG during isolated headache is seldom obtained (Belcastro et al., 2011; Parisi, 2015; Parisi et al., 2012b; Cianchetti et al., 2017a). Only a few confirmed cases, including video-EEG data, support its recognition, suggesting underdiagnosis due to the rarity of performing EEG during isolated headache complaints (Kim et al., 2016; Bauer et al., 2021).

Among seizure-related headache syndromes, postictal headache (PIH) is the most prevalent, affecting a considerable proportion of individuals with epilepsy (Wang et al., 2014b; Kim et al., 2016; Mameniškienė et al., 2016; Çilliler et al., 2017; Mutlu, 2018; Whealy et al., 2019). According to the ICHD-3, PIH is characterized by headache onset within 3 hours following a seizure and spontaneous resolution within 72 h (Headache Classification Committee of the International Headache Society, 2018). Approximately half of those affected describe migraine-like symptoms. Meta-analytic data suggest that one-third of people with epilepsy develop PIH, with postictal migraine occurring in about 16% of cases (Subota et al., 2019). This type of headache is more frequently observed following generalized tonic–clonic seizures than non-convulsive events (Karaali-Savrun et al., 2002) and appears to be more common in patients with occipital lobe epilepsy compared to those with temporal or frontal foci (Wang et al., 2014a). Several risk factors have been identified, including younger age, longer epilepsy duration, higher seizure severity or frequency, and polytherapy with antiseizure medications (Ekstein and Schachter, 2010; Mainieri et al., 2015). Despite its high prevalence, PIH often receives limited clinical attention, as both patients and clinicians tend to focus primarily on seizure control. Consequently, symptomatic treatment with analgesics is frequently overlooked (Paungarttner et al., 2024).

Several pediatric epilepsy syndromes present with clinical and electrographic features that overlap with migraine, particularly in occipital and centrotemporal epilepsies (Nye and Thadani, 2015; Paungarttner et al., 2024). These forms of childhood epilepsy may include headache as part of the seizure presentation, further complicating the clinical picture. Hence, sometimes in adolescence and adulthood, distinguishing between migraine aura and focal seizures could be a clinical challenge, also considering that migraine often begins in childhood or adolescence. Occipital lobe epilepsy shows the strongest association with headache across three distinct forms. In childhood occipital visual epilepsy (previously named Gastaut syndrome), seizures typically begin in the first decade with visual symptoms such as scintillating scotomas or complex hallucinations, occasionally evolving into focal motor seizures. Moreover, postictal migrainous headaches are common. The syndrome usually remits spontaneously. EEG reveals interictal occipital spikes enhanced by eye closure and ictal discharges with anterior propagation (Gastaut, 1982). Self-limited epilepsy with autonomic seizures (previously named Panayiotopoulos syndrome) also presents with occipital EEG abnormalities but is clinically dominated by autonomic symptoms (nausea, vomiting, pallor, lethargy, syncope), impaired consciousness, and migraine-like headaches. Seizures are infrequent and often prolonged (Panayiotopoulos, 1989). Lesional occipital lobe epilepsy features structural brain abnormalities with seizures often beginning with visual auras, then evolving based on propagation patterns (motor or automatisms). Postictal headaches are particularly frequent in this subtype (Williamson et al., 1992). A higher prevalence of migraine has also been observed children with Self-limited Epilepsy with Centrotemporal Spikes (previously known as benign rolandic epilepsy); higher prevalence of migraine is observed also in their relatives suggesting shared genetic susceptibility (Nye and Thadani, 2015). Studies have documented a bidirectional relationship between the two disorders: for instance, children affected by migraine with aura have a higher risk of developing unprovoked seizures, and up to 25% of pediatric epilepsy patients also report migraine. Postictal headaches with migrainous features are frequently reported, especially in self-limited focal epilepsies, where they affect up to 40% of cases (Schiller et al., 2023). Finally, migraine with brainstem aura, a subtype more frequent in children but also observed in adults, is characterized by a constellation of symptoms indicative of transient dysfunction of the brainstem and posterior circulation territories. These include dysarthria, vertigo, tinnitus, diplopia, paresthesia, gait instability (ataxia), and altered consciousness. EEG may reveal features suggestive of encephalopathy or occipital epileptiform abnormalities (mainly irregular high amplitude delta activity) (Lapkin et al., 1977; Camfield et al., 1978; De Romanis et al., 1993). Prophylactic treatment with antiepileptic drugs or calcium channel blockers may be beneficial.

These overlapping phenotypes underline the importance of detailed clinical history and EEG analysis in distinguishing between migraine and epilepsy. EEG is a key tool in the evaluation of epilepsy, with interictal recordings often showing epileptiform abnormalities such as spikes or sharp waves, reflecting cortical hyperexcitability. However, these findings may be absent in patients with infrequent seizures or deep epileptogenic foci. In such challenging cases, conventional scalp EEG presents significant technological gaps in its diagnostic utility. In contrast, EEG is usually normal in primary headache disorders. In migraine, especially with aura, nonspecific changes such as transient focal (temporal or frontotemporal) or diffuse slowing (11%–74% of patients), asymmetries, or enhanced photic driving (the so-called “H response”) have been reported, but lack diagnostic specificity (Smyth and Winter, 1964; Puca and De Tommaso, 1999). Differentiating migraine aura from focal seizures may require ictal EEG, particularly in cases with brief, stereotyped, or evolving symptoms (Italiano et al., 2011). However, despite its diagnostic utility in epilepsy, EEG is not routinely recommended in the evaluation of primary headache disorders. According to the latest EFNS guidelines, its use is reserved for cases in which clinical features raise a suspicion of epilepsy requiring confirmation or exclusion, such as in basilar-type migraine, hemiplegic migraine, or seizure-associated headaches (Sandrini et al., 2011).

### 3.4 Therapeutic intersection

The traditional therapies for migraine prophylaxis include several drugs with different mechanisms of action, including tricyclic antidepressants, beta-blockers, calcium-channel blockers and ASMs (Ornello et al., 2025). ASMs represent one of the most frequently used first-line options for migraine prophylaxis, specifically in patients with comorbid epilepsy (Bauer et al., 2021). However, their exact mechanisms of action in migraine prevention remain poorly understood (Rollo et al., 2023). ASMs stabilize neuronal membranes, modulate neurotransmission, and counteract CSD, potentially reducing central sensitization and offering dual benefits for both seizures and pain relief (Johannessen, 2008). Furthermore, ASMs could reduce neuronal firing and pro-inflammatory release of factors such as calcitonin gene-related peptide (CGRP), a neuropeptide playing a crucial role in pain signaling (Goadsby et al., 2017). The discovery of this novel target within the trigeminovascular system has significantly advanced the understanding of migraine pathophysiology and revolutionized migraine therapy (Ashina, 2020). Moreover, the role of CGRP has also been postulated in epileptogenesis by promoting excitotoxic death of hippocampal neurons in kainate-induced seizure models; however, this finding remains to be confirmed in humans (Park et al., 2013). Table 1 provides an overview of the main ASMs with evidence supporting their use in migraine prophylaxis. Topiramate (TPM) and valproic acid (VPA) are the only two ASMs approved for migraine prophylaxis due to the highest-quality evidence (Ornello et al., 2025). TPM influences sodium and calcium channels, potentiates GABA-A inhibition, and antagonizes AMPA/kainate and NMDA glutamate receptors (Linde, et al., 2013a; Storey et al., 2001). It also inhibits trigeminovascular CGRP release and CSD propagation, significantly reducing migraine days at doses of 100–200 mg/day (Silberstein et al., 2004). Its use may be limited in women of childbearing age, due to potential teratogenicity (Pack et al., 2024). VPA blocks voltage-dependent sodium channels, increases brain GABA levels, and modulates histone deacetylases (Rollo et al., 2023). Clinical trials at 500–1,000 mg/day demonstrate noninferiority to propranolol and near parity with TPM in migraine prevention, though its side-effect profile and teratogenic risk limit its use in young adults with childbearing potential (Linde et al., 2013b; Ashina et al., 2021; Zeinhom et al., 2025). Lamotrigine acts by blocking voltage-gated sodium channels and suppressing glutamatergic transmission, with an action also in inhibiting CSD and visual aura (Buch and Chabriat, 2019); however, this does not yield a reduction in overall headache days versus placebo or TPM in randomized trials (Lampl et al., 2005; Gupta et al., 2007). Levetiracetam binds SV2A synaptic vesicle proteins and blocks N-type calcium channels, showing promising reductions in migraine frequency and intensity in small cohorts, also in children and adolescent populations (Montazerlotfelahi et al., 2019). Perampanel, a selective non-competitive AMPA receptor antagonist, blocks glutamate-mediated excitation and probably inhibits the stimulated release of CGRP from trigeminal neurons (Tringali et al., 2018). In a study enrolling patients with migraine and epilepsy, adjunctive perampanel was associated with a significant reduction in both monthly migraine days and seizure frequency, suggesting its dual modulatory effects on cortical excitability and trigeminovascular signaling (Fernandes et al., 2021). Other ASMs (e.g., gabapentin, pregabalin, lacosamide, zonisamide, carbamazepine and derivates) exhibit variable efficacy in migraine prevention and lack formal approval for this indication (Linde et al., 2013b). Their off-label use, primarily supported by small, observational studies, is rare nowadays since the introduction of novel anti-CGRP monoclonal antibodies and gepants (Ornello et al., 2025). The available evidence on ASMs in migraine prophylaxis remains limited by heterogeneous study designs, insufficient data on long-term safety, tolerability, optimal dosing, and adequate representation of specific populations, such as women of childbearing age and patients with comorbidities.

**TABLE 1:** Main anti-seizure medications used in episodic and chronic migraine prophylaxis.

ASM	Mechanism of action	Studies in migraine prophylaxis (suggested dose)	Main side effects and limitation
Approved for migraine			
Topiramate	- Na^+^ channels block - GABA-A-mediated inhibition enhancement - AMPA/kainate receptors antagonism - NMDA glutamate receptors antagonism - CGRP release inhibition	RCTs and meta-analysis demonstrating efficacy (100–200 mg/day) (Linde et al., 2013b)	Teratogenicity (limitation in women of childbearing age), cognitive side effect, weight loss, kidney stones
Valproic acid	- Na^+^ channels block - GABA-A-mediated inhibition enhancement - Histone deacetylases modulation	RCTs and meta-analysis demonstrating efficacy (500–1,500 mg/day) (Linde et al., 2013c)	High teratogenicity (limitation in women of childbearing age), weight gain, tremor, ataxia
Off label use			
Lamotrigine	- Na^+^ channels block - Glutamatergic transmission inhibition	Two RCTs failing in demonstrating effectiveness (Lampl et al., 2005; Gupta et al., 2007) Four non-randomized trials and case series demonstrating benefit (mainly in migraine with aura; 100–400 mg/day) (Buch and Chabriat, 2019)	Steven-Johonson syndrome, skin rash, dizziness
Perampanel	- AMPA receptor block - CGRP release inhibition	One clinical observational multicentric study showing efficacy (Fernandes et al., 2021)	Somnolence, dizziness, aggression, behavioural disorders
Zonisamide	- Na^+^ channels block - T-type Ca^++^ channels block - CGRP release inhibition	One RCT and one observational study showing effectiveness (Drake et al., 2004; Mohammadianinejad et al., 2011) One observational study demonstrating lack of beneficial effect (Ashkenazi et al., 2006)	Ataxia, fatigue, weight loss, cognitive slowing, kidney stones

AMPA, α-Amino-3-hydroxy-5-methyl-4-isoxazolepropionic acid; AVB, atrioventricular block; Ca^++^, calcium; CGRP, Calcitonin Gene-Related Peptide; GABA, Gamma-Aminobutyric Acid; Na^+^, sodium; NMDA, N-Methyl-D-Aspartate; SV2A, synaptic vesicle protein 2A.

Beyond pharmacotherapy, ketogenic diet and vagus nerve stimulation (VNS) are two diverse approaches further corroborating the therapeutic overlap between migraine and epilepsy (Song et al., 2023). The ketogenic diet enhances mitochondrial energy efficiency, restores neuronal excitability, and probably reduces neuroinflammation in migraine brain, modestly reducing intensity and frequency of headache attacks (Barbanti et al., 2017; Stanton, 2024). In contrast, the ketogenic diet has emerged as a safe and effective therapeutic option for drug-resistant epilepsy by promoting a metabolic shift toward ketone body utilization and enhancing neuronal stability. However, its underlying neural mechanisms remain unclear, and its clinical application remains challenging (Chamma et al., 2025). Vagus nerve stimulation, approved as an adjunctive therapy for drug-resistant epilepsy, exerts its effects by activating central neuromodulatory circuits, including pathways involved in pain modulation and cortical excitability (Cocores et al., 2025). There is a growing interest in its potential application beyond epilepsy, particularly in the treatment of migraine and other primary headache disorders, possibly by influencing trigeminovascular pathways and altering central pain processing (Tassorelli et al., 2018). Finally, Transcranial Magnetic Stimulation (TMS) has emerged as a non-invasive neuromodulation technique for both epilepsy and migraine. In epilepsy, low-frequency TMS (≤1 Hz) is primarily used to reduce cortical excitability and suppress interictal epileptiform discharges, particularly in focal epilepsy (Tergau et al., 1999). In migraine with aura, single-pulse TMS applied to the occipital cortex has proven effective in interrupting CDS (Lipton et al., 2010). This evidence further supports the therapeutic overlap between the two disorders, demonstrating that TMS can modulate the shared abnormalities in cortical excitability and network connectivity. Despite their potential, these non-pharmacological strategies present practical limitations, such as high cost, restricted accessibility, the surgical invasiveness of VNS, and issues related to patient compliance.

## 4 Conclusion

This review has examined the epidemiological, pathophysiological, and clinical intersections between migraine and epilepsy, emphasizing the importance of accurate differential diagnosis and the therapeutic implications driven by shared molecular mechanisms. A growing body of evidence supports a complex and bidirectional relationship between the two disorders. Migraine and epilepsy exhibit significant overlap at epidemiological, pathophysiological, and genetic levels. Shared mechanisms such as cortical hyperexcitability, ion channel dysfunction, and astrocytic impairment support the hypothesis of a common neurobiological substrate. Clinically, their overlapping semiology, especially in aura phenomena, poses significant diagnostic challenges, particularly in pediatric and transitional-age populations. From a therapeutic perspective, the efficacy of several ASMs in migraine prophylaxis highlights shared neurochemical targets and opens the door to integrated treatment strategies. Clinician awareness of the coexistence of both diseases is essential for accurate diagnosis and tailored treatment selection based on individual patient profiles. Nevertheless, key questions remain regarding causality, directionality, and individual susceptibility. Robust and prospective studies, with a focus on underlying mechanisms, are essential to disentangle the nuances of this association and inform personalized diagnostic and therapeutic approaches. Future research should focus on the identification of biomarkers to enhance diagnostic accuracy and the development of novel treatment options, such as neuromodulation techniques, as potential adjunctive therapies.
